# Enhancing Nerve Function and Reviving the Unrecordable: A Comprehensive Analysis of the Effects of a Dual Hydrodissection Protocol on Clinical and Nerve Conduction Parameters in Mild-Moderate and Severe Carpal Tunnel Syndrome

**DOI:** 10.7759/cureus.75681

**Published:** 2024-12-13

**Authors:** Ajay Panwar, Ujjawal Roy, Achal Kumar Srivastava, Pankaj N Surange, Praveen Gupta

**Affiliations:** 1 Neurology, Rotary Ambala Cancer and General Hospital, Ambala Cantt, IND; 2 Neurology, Pulse Hospital, Ranchi, IND; 3 Neurology, Roy Neuro Care Centre, Ranchi, IND; 4 Neurology, All India Institute of Medical Sciences, New Delhi, New Delhi, IND; 5 Pain Management, Interventional Pain and Spine Center, Delhi, IND; 6 Pain Management, Advance Pain Clinic, Alwar, IND

**Keywords:** carpal tunnel syndome, interventional pain medicine, median neuropathy, nerve conduction studies (ncs), nerve hydrodissection, nerve ultrasound, peripheral entrapment neuropathy

## Abstract

Objective: This study evaluates the efficacy of a sequential dual one-month interval hydrodissection (HD) protocol in mild-moderate as well as severe carpal tunnel syndrome (CTS) cases, with a comprehensive analysis of clinical and electrophysiological outcomes.

Methods: A retrospective analysis was conducted on 286 patients (379 wrists) treated between 2021 and 2024 at two centers in India. The enrolled patient population was divided into mild-moderate and severe CTS subgroups. The dual HD protocol comprised an initial HD with methylprednisolone acetate followed by another with 5% dextrose in water (D5W) one month later. Clinical and electrophysiological characteristics were assessed and compared at baseline and three months after initiating the treatment. Outcomes were measured in terms of Visual Analog Scale (VAS) scores, Boston Carpal Tunnel Questionnaire (BCTQ) scores, and nerve conduction studies (NCS) parameters.

Results: The number of patients categorized into mild-moderate and severe subgroups was 239 (321 wrists) and 47 (58 wrists), respectively. Significant improvements were noted among both subgroups. In the mild-moderate subgroup, mean VAS scores decreased from 2.03 to 0.12 (p<0.001). BCTQ functional and severity scores also showed a significant improvement (p<0.001). In the severe subgroup, mean VAS scores decreased from 7.43 to 2.12 (p<0.001), and BCTQ scores also decreased significantly (p<0.001). The key highlight of the study was significant electrophysiological improvement, with 71% of the severe CTS patients showing recordable sensory nerve action potentials (SNAPs) that were non-recordable at baseline.

Conclusion: The dual HD protocol was efficacious in improving clinical as well as electrophysiological outcomes in both mild-moderate and severe CTS patients. This study presents the dual HD strategy as a potentially effective minimally invasive alternative to surgery in severe cases where surgical intervention is the conventional treatment. Further randomized controlled trials with a longer follow-up period are warranted to validate these findings.

## Introduction

Carpal tunnel syndrome (CTS) is the most prevalent focal entrapment neuropathy, comprising almost 90% of all neuropathy cases [1]. This condition results from the compression of the nerve as it passes through the carpal tunnel in the wrist. It is characterized by symptoms such as pain, tingling, and numbness in the distribution of the median nerve, which may result in permanent sensory deficit and loss of motor function if not treated on time [1]. The pathophysiology of CTS involves mechanical compression in the osteofibrous carpal tunnel, which contains the wrist bones, transverse carpal ligament, median nerve, and flexor tendons of hand muscles. The resultant edema and tendon inflammation can further increase compression, thus causing clinical manifestations [2].

The first-line management for mild to moderate cases often includes wrist splinting and non-steroidal anti-inflammatory drugs [3,4]. However, these measures often fail to provide a lasting solution. Quite frequently, corticosteroid injections are combined with splinting, which has shown some superiority compared to splinting alone during the follow-up periods [5]. These injections can rapidly alleviate symptoms by reducing carpal tunnel edema and inflammation and also have membrane-stabilizing and analgesic effects [6,7]. Despite their effectiveness, the benefits of corticosteroid injections are typically short-lived. The injections may need to be repeated frequently, which may be associated with potential side effects [7].

Hydrodissection (HD) is a technique in which fluid injection is used to separate the nerve from surrounding fibrous tissue and adhesions. It has emerged as a modality of treatment for CTS in recent years [8]. This method tends to reduce mechanical compression on the median nerve by creating a physical zone of separation around the nerve. It may potentially enhance the delivery and efficacy of drugs in the perineural space. So far, most of the published literature has shown significant improvement with HD in mild to moderate CTS only, while carpal tunnel release surgery may be necessary in severe cases [8-10]. Our clinical experience suggests that even severe cases of CTS may benefit from this intervention as long as the gross motor deficit or atrophy of the abductor pollicis brevis (APB) muscle has not yet set in. Based on these observations, we aimed to study the clinical and electrophysiological outcomes of HD in mild to moderate as well as severe cases.

This study is based on a dual-hydrodissection protocol, utilizing an initial HD with methylprednisolone acetate followed by another after one month with 5% dextrose in water (D5W). The rationale for this approach is based on the hypothesis that D5W, as a secondary HD agent, might enhance the decompressive effects subsequent to the potent anti-inflammatory effects initiated by the corticosteroid. D5W is an isotonic fluid and is rapidly metabolized, leaving free water, which facilitates the separation of the median nerve from surrounding structures by reducing local edema. This can potentially reduce nerve compression. These osmotic properties of D5W are known to have anti-edema and anti-fibrotic effects, likely resulting in durable improvements in nerve function [10].

In the present study, our objective was to assess the efficacy of this dual sequential HD regimen on both clinical outcomes, as measured by the Visual Analog Scale (VAS) and the Boston Carpal Tunnel Questionnaire (BCTQ), as well as on nerve function through detailed nerve conduction studies (NCS). The results of this study could significantly influence future treatment strategies for CTS, providing further evidence for minimally invasive approaches to treat patients at nearly all stages of the condition.

## Materials and methods

Study design

This retrospective study is a collaborative effort between the neurology departments of two distinct hospitals in India: the "Rotary Ambala Cancer and General Hospital" in northern India at Ambala and the "Roy Neuro Care Centre" in eastern India at Ranchi. This study was possible because of the congruous treatment principles of the two centers. Both the participating centers followed similar diagnostic criteria, management plans, and interventional protocols for CTS. We meticulously reviewed the digital medical records maintained by both neurology departments for every patient with carpal tunnel syndrome who presented in the neurology outpatient department (OPD) consecutively from June 2021 to May 2024. The data retrieved from both centers was merged into a single datasheet, which ensured the standardization of the data entry format and variables. The study was conducted in accordance with the ethical considerations of the Declaration of Helsinki. The study was approved by an institutional ethics committee designated for upholding ethical standards.

Grading of severity of CTS

The severity of CTS was classified from "very mild" to "extremely severe" according to the grading system proposed by Bland et al. (Table 1) [11].

**Table 1:** Grading of severity of carpal tunnel syndrome CTS: carpal tunnel syndrome; APB: abductor pollicis brevis; SNAP: sensory nerve action potential.

Grade	Description
0	No neurophysiological abnormality.
1	Very mild CTS was detected only in sensitive tests.
2	Mild CTS: Orthodromic sensory conduction velocity from index finger to wrist < 40 m/s with motor terminal latency from wrist to APB < 4.5 ms.
3	Moderately severe CTS: motor terminal latency > 4.5 ms and < 6.5 ms with preserved index finger SNAP.
4	Severe CTS: motor terminal latency > 4.5 ms and < 6.5 ms with absent SNAP.
5	Very severe CTS: motor terminal latency > 6.5 ms.
6	Extremely severe CTS: surface motor potential from APB < 0.2 mV (peak-to-peak).

Patient selection and exclusion criteria

The records of consecutively presenting patients who were aged 18 years or older and were diagnosed as CTS on the basis of clinical features and nerve conduction studies were screened for enrollment [2]. Of these, those having an "extremely severe" grade of severity and the ones having gross motor weakness and/or atrophy of the APB muscle were not offered HD as a treatment option and were directly referred for the surgical intervention. The clinical signs of gross APB weakness and atrophy were considered red flags for HD treatment. The patients who were of the mild-moderate grade of severity were initially treated with conservative measures, including wrist splintage and analgesics, and were subsequently treated with HD if nonresponsive to conservative treatment. Those who were of "severe" or "very severe" grade were strongly encouraged for surgery as the first-line treatment while at the same time being explained about HD as an alternative treatment method with reasonable chances of treatment failure and subsequent need for surgery. Of them, the ones who did not consider surgery as the initial treatment option were directly treated with HD rather than the conservative measures. All the patients who underwent surgery due to lack of clinical response after HD were excluded from the study. Further, those who met the following criteria were also excluded from the study: those who received wrist injections for CTS six months prior to the initiation of treatment at our hospital; those who underwent surgery for CTS in the past; those who underwent any additional treatment for CTS at any other place after the initiation of treatment at our hospital during the three months follow-up period; those having multiple mononeuropathies or polyneuropathy due to other conditions viz. diabetic polyneuropathy, rheumatoid arthritis, connective tissue disorders, neoplasm associated or paraneoplastic, infectious etiology, vitamin B12 deficiency, monoclonal gammopathies, sarcoidosis, drug-induced neuropathy as with antitubercular therapy; and lastly, those who were lost to follow-up due to any reason.

Clinical data collection

At the first visit, baseline clinical and demographic data were obtained. The details concerning the duration and severity of symptoms, viz., pain, paresthesias, numbness, and weakness in hands, were noted. A thorough evaluation was done for secondary causes of neuropathy, as mentioned in the exclusion criteria. For systematic evaluation of the degree of severity and functional aspects, we utilized two standardized assessment tools at baseline and at the three-month follow-up.

Visual Analog Scale

This is a reliable visual representation and the most frequently used method to assess pain intensity on a 10-point scale from 0 to 10. It is characterized by a 10 cm line marked from 'no pain' at 0 to 'worst possible pain' at 10 [12]. Patients were explained about the scale in their vernacular or spoken language and asked to mark a point on the line corresponding to their current intensity of pain.

Boston Carpal Tunnel Questionnaire

The BCTQ is a validated set of questions for assessment of the severity of symptoms and functional status related to CTS. It has 11 questions on severity and eight questions on functional status. Each question is scored from 0 to 5, where a score of 0 means mildest or no symptoms with no difficulty in activity, while a score of 5 means very serious symptoms and inability to perform the activities [13].

Prior to the administration of questionnaires, a healthcare professional trained in data recording and handling explained the purpose and method of clinical assessments to ensure that the patients understood how to report their severity and functional status correctly. Responses were initially recorded by the same healthcare professional in the printed questionnaires and later transformed into digital data. Patients were also explained about the grades of severity of CTS in an easily comprehensible way, enabling them to properly understand their condition and the treatment methods. This made sure of an educated informed consent.

Nerve conduction studies

A Natus machine with UltraPro™ S100 Synergy software (Natus Medical, Inc., Middleton, WI, USA) was used in all subjects for nerve conduction studies. The temperature of patients’ extremities was kept warm, as colder temperatures can falsely alter test values by prolonging distal latencies and decreasing conduction velocities. A notch filter was used to reduce electrical interference. All nonessential machinery was turned off wherever possible to minimize potential disruptions.

Sensory conduction studies involved stimulation of the median nerve at the wrist and recording the sensory nerve action potential (SNAP) at the base of the second or third finger, depending on the clinical picture. Results were compared with SNAPs of the ulnar nerve (stimulation at the wrist and recording at digit five) and radial nerve (stimulation at the lateral radius and recording at the snuffbox) [14]. Motor conduction studies were recorded from the abductor pollicis brevis muscle. For motor nerve conduction studies, the active and reference electrodes were placed over the abductor pollicis brevis muscle and the first metacarpophalangeal joint, respectively. The median nerve stimulations were given distally at the wrist and proximally at the elbow. Results obtained were compared with age-appropriate standard values and with other nerves of the same hand [15]. Comparative motor testing with the ulnar nerve included recording from the abductor digiti minimi while stimulating at the wrist distally and at the elbow proximally. Auxiliary comparison studies were done for patients who had normal routine NCS in the clinical setting suggestive of CTS. These included comparing the distal motor latencies of the second lumbrical muscle controlled by the median nerve and the interossei muscles controlled by the ulnar nerve. Sensory latencies were compared at the base of the fourth digit by individually stimulating the median and ulnar nerves at the wrist at identical distances [14].

Dual sequential hydrodissection protocol

Patients underwent two sequential HD treatments. Initially, HD was done using 40 mg (1 ml) methylprednisolone acetate with 4 ml of lidocaine. After one month, the second session of HD was performed with 5 ml of a 5% dextrose water (D5W) solution to enhance and sustain the decompressive effect achieved in the first HD session.

Ultrasound-guided hydrodissection technique

The procedure was done by positioning the patient comfortably with the forearm supinated and flexed at 90°, the wrist rested on the couch, and the fingers semi-extended to facilitate comfortable needle access to the carpal tunnel. We used the following make of USG systems at two centers: (i) BPL ECUBE i7 Ultrasound Machine (BPL Medical Technologies, Bengaluru, India) with a Linear Hockey Stick Transducer IO8-17T (8-17 MHz); (ii) Venue Go™ R4 (GE HealthCare Technologies, Inc., Chicago, Illinois, USA) with a 4-20 MHz linear transducer. The pre-programmed settings (viz., depth, focal zone, frequency, and color Doppler settings) of our USG systems helped to enhance the details of the structures of the carpal tunnel and nearby low-flow blood vessels.

A detailed ultrasound examination of the carpal tunnel was performed to identify and assess for any anatomical abnormalities such as ganglion cysts, flexor tendon tenosynovitis, and anatomical variations like a bifid median nerve or a persistent median artery, which may have any potential implications on the procedure.

The HD procedure was performed under strict aseptic conditions. Using an in-plane ulnar approach, a 25-gauge needle was introduced laterally and directed toward the median nerve in the carpal tunnel [16]. The ulnar approach of carpal tunnel HD was taken to avoid injury to the recurrent branch of the median nerve. This branch innervates the thenar muscles and is prone to injury during carpal tunnel interventions [17]. A single operating physician performed the procedure while holding the ultrasound probe with the left hand and the syringe with the right. The needle tip movement was closely observed and precisely controlled using the continuous USG visualization. The injectate was administered slowly, carefully dissecting the flexor retinaculum and flexor tendons away from the median nerve by creating a safe zone of separation from the median nerve. We delivered 2 ml and 3 ml of injectate to separate the median nerve from the flexor tendons and flexor retinaculum, respectively. Video 1 shows the detailed, annotated technique of USG-guided carpal tunnel hydrodissection for CTS. We observed the patients for 30 minutes during the post-procedural period to check for any complications such as swelling, pain, and bleeding, and then discharged them.

**Video 1:** USG-guided carpal tunnel hydrodissection for CTS Detailed annotated technique of USG-guided carpal tunnel hydrodissection for CTS

Statistical analysis

Data analysis was performed using SPSS statistical software version 23.0 (IBM, Armonk, NY). Categorical variables were computed as percentages, and continuous variables as mean ± standard deviation. Paired Student’s t-test was used to compare changes in VAS scores at baseline and three months of follow-up. The Wilcoxon signed-rank test was used to compare changes in BCTQ scores and nerve conduction parameters. P values <0.01 were considered significant.

Subgroup analysis

Patients were stratified into two broad subgroups for analysis based on severity: mild-moderate and severe. The "mild-moderate" subgroup included CTS cases of mild and moderate severity (grades 1-3), while the "severe" subgroup included cases of severe and very severe disease (grades 4-5). Split data analysis was performed based on this stratification to study if this HD protocol could be effective for even severe cases.

## Results

**Figure 1:**
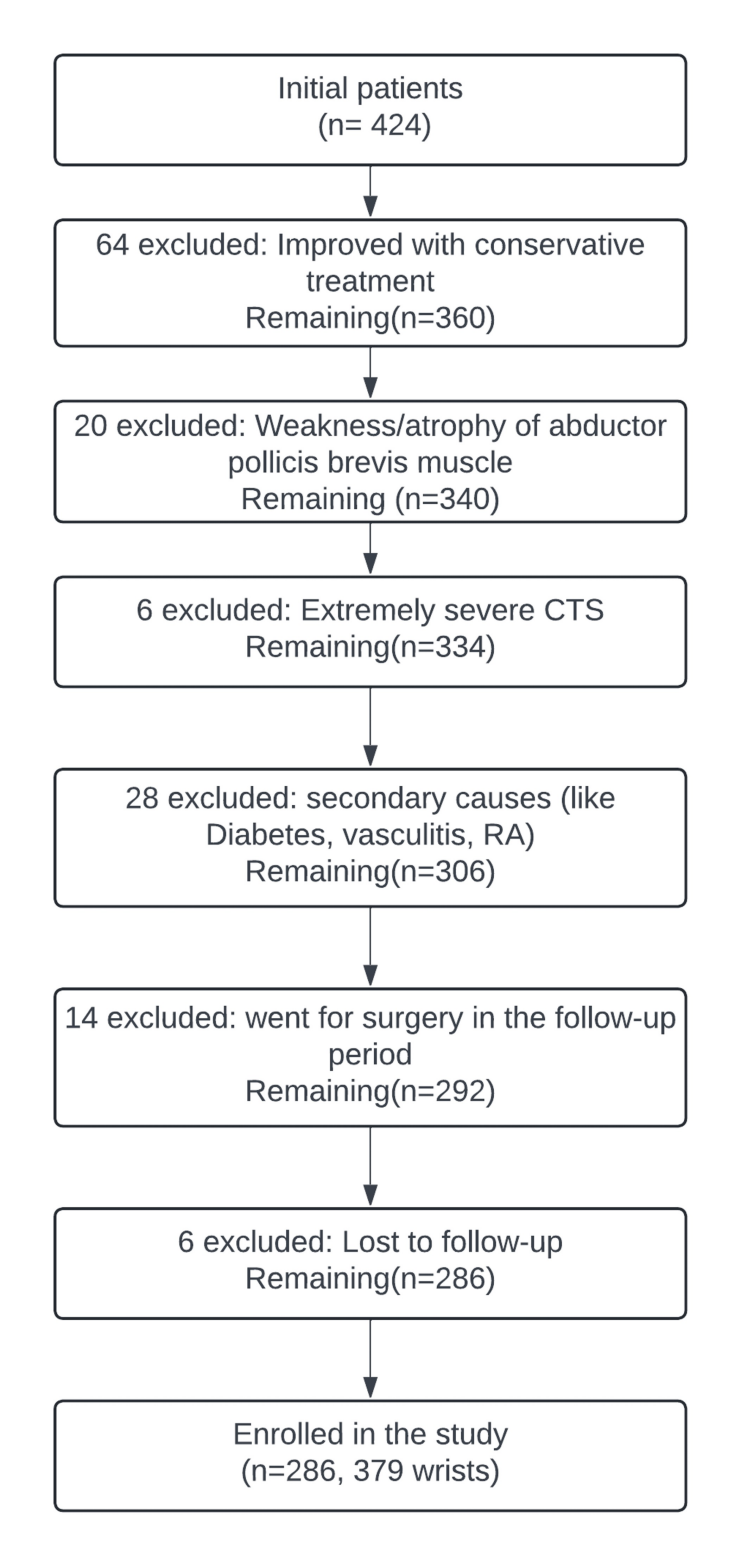
Algorithmic approach to patient selection and exclusion criteria in the study An algorithmic approach to patient selection and exclusion criteria in the study. The flowchart details the stepwise process of patient exclusion and final enrollment.

During the study, 424 patients with CTS were observed in neurology OPDs at the two centers. After applying the exclusion criteria, 286 patients with 379 wrists were ultimately included in the study (Figure 1).

The mean age of the study subjects was 37.34 (±5.48) years, and 74.9% of the enrolled patients were females. Regarding the severity of CTS patients, the distribution of cases across grades 1, 2, 3, 4, and 5 was 10.6% (n=40), 55.1% (n=209), 19% (n=72), 9.2% (n=35), and 6.1% (n=23), respectively. Further categorization placed 239 patients with 321 wrists in the "mild-moderate" (grades 1-3) and 47 patients with 58 wrists in the "severe" (grades 4-5) subgroups.

Comparative analysis of clinical outcomes at baseline and three months

A paired t-test was used to compare the VAS scores at baseline and three months. The mean VAS score in the "mild-moderate" subgroup was 2.03 (±1.72) at baseline and reduced to 0.12 (±0.42) at three months. The difference was statistically significant (p<0.001, 95% CI: 1.74-2.08). Likewise, the mean VAS score in the "severe" subgroup also reduced significantly (p<0.001, 95% CI: 4.92-5.69) from 7.43 (±1.50) at baseline to 2.12 (±0.72) at three months. We could not use the paired t-test for the comparisons of BCTQ-Functional Status Scale (FSS) and Severity Status Scale (SSS) scores because the data were not normally distributed as assessed by the Shapiro-Wilk test of normality. So, we used the Wilcoxon signed-rank test, a non-parametric alternative for these outcomes. Both FSS and SSS scores showed a significant reduction after three months as compared to the baseline. In the "mild-moderate" subgroup, mean FSS scores reduced from 11.87 (±4.1) at baseline to 08.6 (±0.6) at three months (p<0.001), while mean SSS scores reduced significantly as well (p<0.001) from 18.05 (±5.8) to 12.24 (±1.6) (Table 2).

**Table 2:** Comparison of clinical and electrophysiological parameters at baseline and three months after the initial treatment in the mild-moderate subgroup (239 patients, 321 wrists) VAS: Visual Analogue Scale; BCTQ: Boston Carpal Tunnel Questionnaire; FSS: Functional Status Scale, SSS: Symptom Severity Scale; SNAP: sensory nerve action potential; CMAP: compound muscle action potential; SD: standard deviation; CI: confidence interval; ms: milliseconds; µV: microvolts; mV: millivolts. *Student’s paired t-test, ^†^Wilcoxon signed rank test, ^††^VAS score ranges from 0 (no pain) to 10 (worst possible pain). BCTQ score ranges from 1 to 5 per item, with the cumulative score for BCTQ-FSS ranging from 8 to 40 and for BCTQ-SSS ranging from 11 to 55.

	Baseline	3 months	P-value
VAS score ^††^ (mean ± SD)	2.03±1.72	0.12±0.42	<0.001 (95%CI: 1.74–2.08)^*^
BCTQ-FSS^††^	11.87±4.1	08.6±0.6	<0.001^†^
BCTQ-SSS^††^	18.05±5.8	12.24±1.6	<0.001^†^
SNAP			
Peak latency (ms)	03.85±0.33	3.41±0.11	<0.001^†^
Conduction velocity (m/s)	35.78±4.64	43.22±2.2	<0.001^†^
Amplitude (µV)	15.98±3.60	22.95±5.41	<0.001^†^
CMAP			
Distal latency (ms)	04.13±0.29	03.86±0.21	<0.01^†^
Distal amplitudes (mV)	07.82±1.52	08.11±1.46	<0.01^†^
Proximal amplitudes (mV)	07.16±1.46	07.40±1.36	<0.01^†^

In the "severe" subgroup also, both these scores showed a significant (p<0.001) reduction at three months. FSS scores decreased from 28.10 (±3.23) at baseline to 14.62 (±3.27) at three months. Likewise, SSS scores decreased from 39.43 (±3.44) to 19.57 (±4.05) (Table 3).

**Table 3:** Comparison of clinical and electrophysiological parameters at baseline and 3 months after the initial treatment in the severe subgroup (47 patients, 58 wrists) VAS: Visual Analogue Scale, BCTQ: Boston Carpal Tunnel Questionnaire; FSS: Functional Status Scale, SSS: Symptom Severity Scale, CMAP: compound muscle action potential, SD: standard deviation; CI: confidence interval; ms: milliseconds; mV: millivolts. *Student’s paired t-test, ^†^Wilcoxon signed rank test, ^††^VAS score ranges from 0 (no pain) to 10 (worst possible pain). BCTQ score ranges from 1 to 5 per item, with the cumulative score for BCTQ-FSS ranging from 8 to 40 and for BCTQ-SSS ranging from 11 to 55.

	Baseline	3 months	P-value
VAS score ^††^ (mean±SD)	7.43±1.50	2.12±0.72	<0.001 (95%CI: 4.92–5.69)^*^
BCTQ-FSS^††^	28.10±3.23	14.62±3.27	<0.001^†^
BCTQ-SSS^††^	39.43±3.44	19.57±4.05	<0.001^†^
CMAP			
Distal latency (ms)	05.62±0.98	04.52±0.26	<0.01^†^
Distal amplitudes (mV)	03.64±0.90	03.97±0.89	<0.01^†^
Proximal amplitudes (mV)	03.08±0.86	03.35±0.83	<0.01^†^

Comparative analysis of electrophysiological outcomes at baseline and three months

Again, the distribution of electrophysiological parameters did not follow a normal distribution, and the Wilcoxon signed-rank test was used for comparisons at baseline and three months.

Mild-moderate subgroup

In the "mild-moderate" subgroup, SNAPs showed significant improvements in terms of peak latencies, amplitudes, and conduction velocities at three months as compared with baseline (p<0.001). While the apparently observed differences in the "compound muscle action potentials" appeared minimal, they achieved significance levels on statistical analysis (p<0.01) (Table 2).

Severe subgroup

The most striking observation in the "severe" subgroup was that a major proportion (41/58; 71%) of the patients whose SNAPs were unrecordable at the baseline became recordable at three months, albeit with reduced amplitudes. A binomial test confirmed this to be statistically significant (p=0.002). CMAPs also showed significant differences in the distal latencies and amplitudes (p<0.01) (Table 3).

## Discussion

The present study evaluated the clinical and electrophysiological outcomes of a dual sequential one-month interval HD treatment strategy combining methylprednisolone acetate, followed by 5% D5W, in patients across the various severity grades of CTS. Our results support the efficacy of this treatment protocol, especially for severe cases of CTS, where the contemporary management approach often advocates surgical intervention.

This dual HD approach showed significant improvements in pain, as reflected by the VAS. On similar lines, BCTQ scores for severity and functional outcomes also showed significant improvements. These observations are in agreement with the previous studies that proved the effectiveness of HD in pain in mild to moderate CTS cases [18-21]. However, our observations suggest evidence that this technique can also be beneficial in severe cases where surgery is typically recommended. Currently, there are no studies in medical literature to thoroughly evaluate and validate the use of HD in severe CTS with respect to clinical outcomes and detailed electrophysiological observations. The significant improvement observed in the severity of symptoms as well as function in our severe subgroup opens the door for minimally invasive non-surgical interventions in this patient population.

In terms of nerve conduction parameters, we observed significant improvements in both sensory and motor nerve conduction. The unrecordable SNAPs becoming recordable in 71% of severe cases is the high point of this study. This suggests that this dual HD protocol might improve nerve function even in severely compromised nerve conduction. Prior studies have also shown improvements in electrophysiological parameters following HD, though they have predominantly focused on mild-moderate cases [22,23]. Wu et al., in their double-blind, randomized controlled trial (RCT) for saline HD in mild-moderate CTS, observed significant improvement in SNAP conduction velocities at three and six months. Still, improvements in distal motor latencies were not statistically significant. In contrast, we observed statistically significant improvements in distal motor latency as well as CMAP amplitudes. The improvement in CMAPs, while less pronounced than in sensory potentials, suggests that motor recovery is also possible. This concept of motor recovery is also supported by a double-blind controlled RCT by Elawamy et al., who observed significant improvement in distal motor latency with hyalase HD [18]. This strongly supports the hypothesis that HD may promote nerve regeneration, likely by reducing the mechanical compression that blocks nerve conduction [24].

The existing literature suggests that corticosteroid injections alone may offer only short-lasting benefits in severe CTS. The addition of a sequential HD with another agent, in particular with D5W, is likely to provide a more sustained improvement in clinical as well as electrophysiological parameters. Recently, Juan-He et al. also observed significant clinical improvements in terms of VAS and BCTQ scores with a similar dual HD protocol of corticosteroid injection followed by D5W HD after four weeks. They observed sustained clinical improvements up to 12 weeks after the follow-up. However, they did not conduct the electrophysiological evaluations [25]. A step further, with a larger patient population, including severe cases, our study more comprehensively validates their observations as it dives deep into analyzing the neurophysiological recovery with a similar treatment protocol. Conventionally, severe CTS is managed surgically, and non-invasive treatments like splinting and corticosteroid injections are considered for milder cases. The risk of surgical complications, contraindications for surgical treatment in some cases, and patient preference for non-surgical options necessitate the need for effective alternatives. The recovery of nerve conduction in non-recordable nerves may potentially delay or even obviate the need for decompressive surgery. In the advancing era of minimally invasive treatment options, we suggest that the dual HD protocol may offer a viable alternative to surgery.

Despite the promising findings, our study has several limitations. First, it was a retrospective analysis, and the observations need to be tested through RCT to strengthen the conclusions. Further, while the three-month follow-up period is sufficient to establish short-term outcomes, longer-term data are warranted in determining the sustainability of the improvements in clinical as well as electrophysiological parameters. Lastly, we excluded patients with extremely severe CTS (grade 6), thus limiting the validity of the findings to these patient populations. It can be explored in further research whether modifications to the HD protocol, such as using different injectates or multiple HDs at different time frames, might benefit these patients.

## Conclusions

Our study shows that a sequential dual HD protocol combining corticosteroids and D5W is efficacious in improving both clinical and electrophysiological outcomes in patients with CTS of even severe grade. This protocol offers a promising minimally invasive alternative to surgery. Future RCTs with larger patient populations and longer follow-up periods should be conducted to further validate these results and refine the treatment protocol.
